# Periodontitis, Dyslipidemia and Rheumatoid Arthritis: An Additive Model of Cardiovascular Risk

**DOI:** 10.3390/jcm14196722

**Published:** 2025-09-23

**Authors:** Marco Bonilla, Enrique Raya-Álvarez, Manuel Bravo, Eva Rosel, Francisco Mesa

**Affiliations:** 1 Department of Periodontics, School of Dentistry, University of Granada, 18071 Granada, Spain; 2 Department of Medicine, School of Medicine, University of Granada, 18016 Granada, Spain; enriraya@ugr.es; 3 Department of Rheumatology, San Cecilio University Clinical Hospital, 18016 Granada, Spain; 4 Department of Preventive and Community Dentistry, School of Dentistry, University of Granada, 18071 Granada, Spain; mbravo@ugr.es (M.B.); erosel@ugr.es (E.R.)

**Keywords:** periodontitis, cardiovascular risk, dyslipidemia, rheumatoid arthritis

## Abstract

**Background**: Rheumatoid arthritis and periodontitis are chronic inflammatory diseases linked to systemic complications, including increased cardiovascular risk. The impact of glycemia, lipid profile and atherogenic cardiovascular risk indices in patients with rheumatoid arthritis (RA) and periodontitis, compared to controls, has not yet been evaluated. We aimed to analyze whether periodontitis acts as an aggravating factor in this relationship. **Methods**: In a case–control study, we assessed biochemical, RA-related markers and four atherogenic indices (Atherogenic Index of Plasma, Castelli Risk Index I, Castelli Risk Index II, and Triglyceride–Glucose Index). Periodontitis was evaluated using a gingival inflammation index (BOP) and a periodontal severity index (PIRIM). Multiple linear regression models were used to analyze whether periodontitis had a differential effect in RA cases versus controls. **Results:** A total of 46 participants were included (32 RA cases, 14 controls). Periodontitis was more prevalent among cases (62.5% vs. 28.5%). BOP was significantly higher in RA patients (*p* < 0.001) and associated with LDLC (*p* = 0.031). Both BOP and PIRIM correlated with higher CRI-1 and CRI-2 values across groups. PIRIM was also linked to increased LDLC (*p* = 0.018) and decreased HDLC (*p* = 0.003). **Conclusions:** RA and periodontitis appear to interact synergistically and are associated with a more atherogenic profile. These findings highlight periodontal health as a potentially modifiable factor in reducing cardiovascular risk in RA patients.

## 1. Introduction

Rheumatoid arthritis (RA) is a chronic autoimmune inflammatory disease that primarily affects the joints, causing persistent inflammation that can lead to structural damage and functional disability [1]. The etiology remains unknown; however, environmental and behavioral factors along with, more recently, peptide citrullination by periodontal pathogens, are recognized pathogenic contributors [2,3]. Substantial scientific evidence accumulated over the past 25 years indicates that systemic inflammation, along with damage to vascular walls, plays a critical role in the development of atherosclerosis and, consequently, in increased cardiovascular risk [4]. The relative risk of a cardiovascular event in patients with RA is estimated to be double that of age- and sex-matched individuals without RA, independent of the traditional factors of cardiovascular risk [5]. A recent 10-year follow-up study in RA patients without prior cardiovascular events showed that 8.4% of patients experienced a first cardiovascular event, with arterial hypertension being the most frequent [6].

Similarly, periodontitis, a chronic inflammatory disease that affects the supporting tissues of the teeth, presents a multifactorial etiopathogenesis, involving a dysregulated immune response and subgingival dysbiosis, with the elevated production of pro-inflammatory cytokines and tissue-degrading mediators [7]. Mesa et al. summarized in a review the cardiovascular disease risk in periodontal patients, highlighting the presence of periodontal pathobionts such as *Porphyromonas gingivalis*, *Tannerella forsythia* and *Treponema denticola* in atheroma plaques, and how *Porphyromonas gingivalis* can induce platelet aggregation and increase the expression of adhesion molecules in the endothelium, facilitating vascular inflammation and thrombosis [8]. In recent years, periodontitis and RA have been proposed to share common pathological mechanisms, such as peptide citrullination and the overproduction of pro-inflammatory cytokines like IL-6 and TNF-α [9], both contributing to chronic systemic inflammation and endothelial dysfunction. Consequently, each disease is considered an independent risk factor for cardiovascular events. Periodontitis has a bidirectional relationship with diabetes, adding further burden in vascular risk, immune dysfunction and tissue damage [10].

Parameters such as glycemia and atherogenic profile with cardiovascular risk indices have been studied in patients with RA [11] and periodontitis [12] separately, but the relationship between these parameters in patients with both RA and periodontitis, compared to controls, has not yet been established. We hypothesize that patients with RA and periodontitis exhibit higher atherogenic indices of cardiovascular risk in comparison with those without periodontitis, which suggests a possible synergistic interaction between the two diseases.

This study aimed to evaluate biochemical and glycemic variables, as well as four atherogenic cardiovascular risk indices, in RA patients compared with non-RA controls, analyzing periodontitis as a potential aggravating factor in this relationship.

## 2. Materials and Methods

### 2.1. Study Design and Participants

An observational case–control study was carried out between July 2024 and February 2025. The case group consisted of patients diagnosed with RA, while the control group included healthy individuals or patients with non-inflammatory degenerative joint conditions. Participants were recruited from the Rheumatology Department of the San Cecilio University Clinical Hospital (Granada, Spain). Inclusion criteria for the RA group were adult patients with a confirmed diagnosis of RA based on the criteria of the American College of Rheumatology (ACR) [13]. Controls were healthy adults or individuals with non-inflammatory musculoskeletal conditions from the same clinical setting. Exclusion criteria for both groups included having received periodontal treatment within the past year, being edentulous or having fewer than 10 teeth, presenting with any systemic inflammatory condition other than those under study, immunodeficiency disorders, or pregnancy. In addition, control participants who had taken antibiotics and/or anti-inflammatory medications within the previous two months were excluded.

This study was conducted in accordance with the principles of the Declaration of Helsinki, 2013 revision. The methodological design adhered to the Strengthening the Reporting of Observational Studies in Epidemiology (STROBE) guidelines for observational research [14], and the completed checklist is provided in the Supplementary Materials. All participants provided verbal informed consent after being informed of the study’s purpose and procedures. The study protocol was approved by the Ethics Committee of the Provincial Research Committee of Granada, Spain (registration number: SICEIA-2024-002311).

### 2.2. Study Variables

Patient Medication: RA patients followed standard treatment protocols, including daily doses of Prednisone (2.5–7.5 mg), weekly Methotrexate (10–22.5 mg)—a conventional synthetic DMARD—and/or biweekly Adalimumab (40 mg)—a biologic DMARD.

Sociodemographic Variables:

Age, gender and tobacco consumption in cigarettes per day.

Periodontal Variables:

Probing pocket depth (PPD), measured in millimeters, and bleeding on probing (BOP), expressed as a percentage [15], were used as diagnostic parameters for periodontitis. The total number of present teeth was also recorded. The severity of periodontitis was assessed using the PIRIM, calculated as the sum of all periodontal pockets > 4 mm, normalized by the number of remaining teeth [16]. The diagnostic criteria for periodontitis followed the 2018 classification by the American Academy of Periodontology and the European Federation of Periodontology [17], defined as the presence of interdental clinical attachment loss (CAL) ≥ 3 mm at ≥2 non-adjacent teeth, with PPD ≥ 4 mm and BOP.

All measurements were performed by a single calibrated examiner (M.M.) using a PCP-UNC15 manual periodontal probe (Hu-Friedy, Chicago, IL, USA). Prior to data collection, examiner M.M. was calibrated by the reference investigator (F.M.) for inter- and intra-examiner reliability in measuring PPD and CAL at the School of Dentistry, University of Granada. Concordance levels were 79% and 82%, respectively, with a ±1 mm variation considered acceptable. In partially edentulous individuals, removable prostheses were taken out during examination. In the case of fixed restorations, only natural teeth were probed, and implants were excluded.

Biochemical Variables:

The most recent laboratory tests included the measurement of the following biomarkers: rheumatoid factor (RF), expressed in international units per milliliter (IU/mL); anti-citrullinated peptide antibody (ACPA), expressed in units per milliliter (U/mL); and C-reactive protein (CRP), quantified by enzyme-linked immunosorbent assay (ELISA) and reported in milligrams per liter (mg/L). Additionally, the lipid profile was assessed, including total cholesterol, low-density lipoprotein cholesterol (LDL-C), high-density lipoprotein cholesterol (HDL-C) and triglycerides, all expressed in milligrams per deciliter (mg/dL). Fasting glucose levels were also recorded in mg/dL. For patients with RA, laboratory tests are routinely performed every 3–6 months depending on clinical symptoms, whereas for healthy controls, only results obtained within the past year were considered.

**Figure 1:**
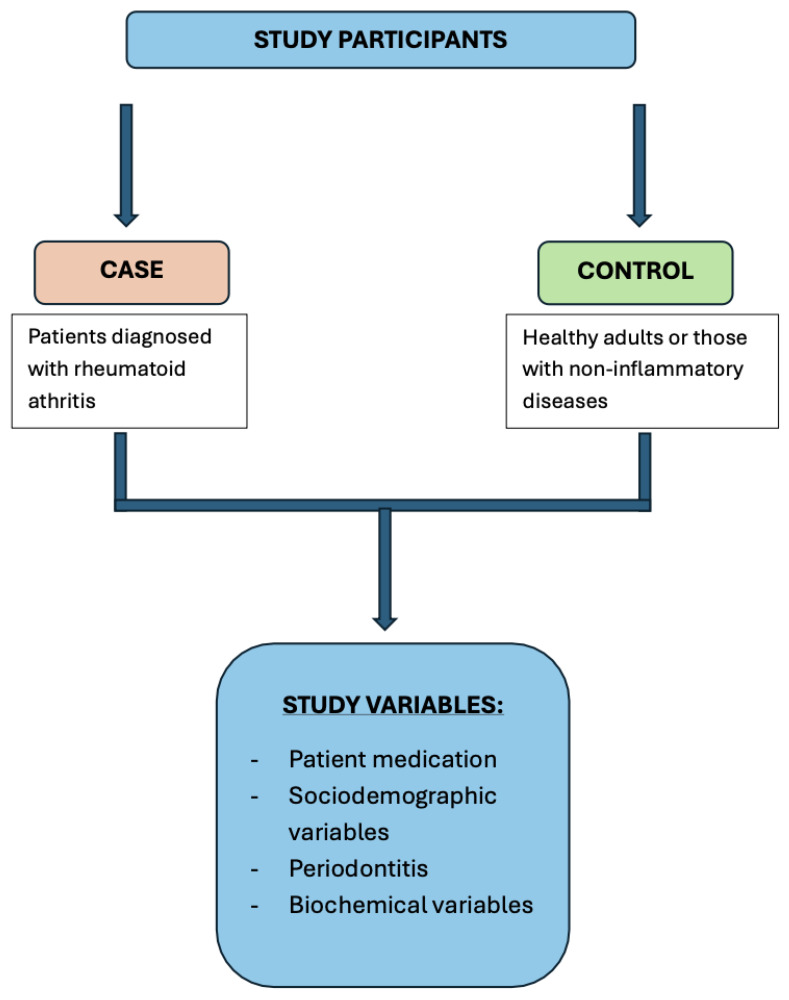
Graphical flowchart of the experimental design.

The experimental design and patient flow are summarized in Figure 1.

### 2.3. Atherogenic Indices

The Atherogenic Index of Plasma (AIP) was calculated as the base-10 logarithm of the ratio between triglycerides and HDL cholesterol, both expressed in mmol/L: AIP = log_10_ (TG/HDL-C). According to established thresholds [18], AIP values were classified as low risk: <0.1, intermediate risk: 0.1–0.24, and high risk: >0.24.

The Castelli Risk Index I (CRI-1) was calculated as the ratio of total cholesterol to HDL cholesterol (TC/HDL-C), with values < 3.5 indicating low cardiovascular risk. The Castelli Risk Index II (CRI-2) was calculated as the ratio of LDL cholesterol to HDL cholesterol (LDL-C/HDL-C), with values < 3.0 indicating low cardiovascular risk [19].

The Triglyceride–Glucose Index (TyG index) was calculated as TyG = Ln [Triglycerides (mg/dL) × Glucose (mg/dL)/2]. The cut-off value for identifying the risk of heart failure was ≥8.9 [20].

### 2.4. Statistical Methods

The calculation of statistical power for this analysis was estimated following a general and standardized rule, with the help of Sample Power 2.0 (SPSS Inc., Chicago, IL, USA). Thus, for a sample size of *n* = 39, with an effective n for any quantitative variable (i.e., cardiovascular risk variables CRI 1 and CRI 2, periodontal variables as BOP, etc.) divided into two groups (10 arthritis controls and 29 cases), it is possible to detect, with 80% power and a 5% alpha error, a standardized difference, using the *t* test, in quantitative variables of 1.0, which is close to substantial according to Cohen’s scale [21].

The statistical analysis was performed with IBM SPSS Statistics 22.0 (IBM Corp., Armonk, NY, USA), using descriptive and analytical methods specified in each results table. To analyze whether the periodontal variables, BOP and PIRIM, could have a different effect in cases and controls, multiple linear regression models were used, including the interaction with the case variable. Except for one instance, this interaction was not significant, and the models were built by forcing the variables case and BOP/PIRIM and testing the inclusion of sex or age based on their statistical significance. However, in no case did these variables enter the models (see Table 3). Note that this statistical analytical strategy is within the recommended limits for multivariate data analysis (e.g., regression analysis), i.e., the sample size should be 10 times greater than the number of variables [22].

## 3. Results

A total of 46 participants were included in this case–control study, 32 cases and 14 controls. Among the cases, 20 (62.5%) had periodontitis, compared with 4 (28.5%) in the controls. Table 1 shows the comparison of sociodemographic and periodontal variables between both groups. Gingival inflammation, measured by BOP, was significantly higher in cases compared with controls (*p* < 0.001).

Table 2 compares the included biochemical variables and atherogenic indices, showing that both groups exhibited comparable values, except for the markers (ACPA and RF) that define the case group.

Table 3 presents a total of 36 multiple linear regression models, i.e., 18 models including BOP as the predictor variable and 18 models including PIRIM, with both BOP and PIRIM as proxy variables of periodontitis. We built, for each BOP or PIRIM, two models for each dependent variable (glycemia, etc.). The first models (type I) analyze the eventual interaction between case/control and BOP/PIRIM, and it only tested significantly (*p* = 0.031) for case/control × BOP against LDLC. The type II models forced the variables case/control, BOP/PIRIM, age and sex, but we only present the coefficient (β ± se) and *p*-value for the periodontal proxy variable (i.e., BOP or PIRIM). Thus, BOP is significantly associated with LDLC, CRI-1 and CRI-2, and PIRIM with LDLC, HDLC, CRI-1 and CRI-2 (Table 3), with the presence of that periodontal proxy variable being associated with higher values of those dependent variables, except for HDLC with PIRIM.

## 4. Discussion

Our study demonstrates that periodontal inflammation (measured as BOP) and severity (measured as PIRIM) are associated with a higher cardiovascular risk, measured by LDLC, HDLC, CRI-1, CRI-2 and a near-significant association between PIRIM and AIP. The interaction between case × BOP with LDLC was statistically significant, while no other significant interaction was observed between BOP and PIRIM with other variables. This suggests that these periodontal parameters are consistently associated with higher lipid and atherogenic indices across both RA patients and controls.

Periodontal pathobionts, via lipid oxidation, can induce systemic dyslipidemia [23], characterized by elevated LDLC and reduced HDLC. This lipid imbalance is linked to an increased risk of atherosclerotic cardiovascular diseases, as high LDLC levels are a well-established risk factor for atheromatous plaque accumulation in arteries [24], while a decrease in HDLC impairs reverse cholesterol transport, thereby promoting cholesterol build-up in arterial walls [25]. In this context, we observed a statistically significant interplay between BOP and LDLC (*p* = 0.031), which indicates that BOP is associated with greater LDLC levels only in the RA patient group. Additionally, BOP exhibited a negative tendency (*p* = 0.084) towards HDLC levels in both groups, further emphasizing the association between periodontal inflammation and dyslipidemia.

In our study, BOP showed a statistically significant association with atherogenic indices CRI-1 and CRI-2 (*p* < 0.001) in both study groups. According to their formulas, an increase in CRI-1 and CRI-2 values indicates a higher ratio of total cholesterol and LDL relative to HDLC, which is recognized as a risk factor for atherosclerosis. Li et al. identified CRI-1 and CRI-2 as predictors of coronary artery disease, suggesting that their association with BOP implies that periodontal inflammation, even in control patients, correlates with a profile of patients with a higher coronary risk [26]. Although the association between BOP and total cholesterol did not reach statistical significance (*p* = 0.068), other authors have reported an association between periodontitis and increased total cholesterol levels [27]. These findings support the hypothesis that periodontal inflammation, independent of the RA condition, is associated with cholesterol and acute-phase protein secretion in the liver, fostering a low-grade pro-inflammatory environment that supports the development of an atherogenic systemic profile.

PIRIM is an index reflecting the cumulative severity of periodontal damage. It is calculated as the ratio of the sum of all periodontal pockets ≥ 4 mm normalized by the number of remaining teeth. Our findings show a non-significant trend towards an association between PIRIM and the TyG index exclusively in RA patients (*p* = 0.093). The TyG index, an indicator of the ratio between triglyceride and fasting glucose levels, has been associated with a higher risk of cardiovascular diseases, including myocardial infarction [20]. Furthermore, we observed a statistically significant negative association with HDLC levels (*p* = 0.003) in both groups, consistent with the previously discussed lipid imbalance. Low HDLC levels are an independent risk factor for cardiovascular diseases, as HDLC plays a pivotal role in reverse cholesterol transport, facilitating the removal of excess cholesterol from arterial walls [25]. This association suggests that the progression of periodontitis over time, reflected in a greater number and/or depth of periodontal pockets, is associated with an increased cardiovascular risk in both RA and healthy patients.

Similarly to BOP, PIRIM also exhibited positive associations with CRI-1 and CRI-2 (*p* < 0.001 for both groups). This finding is consistent with the fact that both variables are integral indicators of periodontitis, reflecting similar clinical implications. Although the relationship between PIRIM and AIP did not reach statistical significance (*p* = 0.057), the observed trend suggests that increased cumulative periodontal destruction could be linked to a more atherogenic lipid profile. The AIP serves as a marker that reflects the balance between triglyceride-rich lipoproteins and HDLC and is correlated with an increased risk of myocardial infarction and other cardiovascular diseases [28]. Elevated AIP values have been associated with additional cardiovascular risk factors, including hypercholesterolemia, hyperuricemia, metabolic syndrome and metabolic diseases such as hypertension and diabetes [29]. While CRI-1 and CRI-2, with which we found statistically significant associations, focus on the ratio of total cholesterol or LDL to HDL, AIP provides complementary insights into triglyceride and LDL metabolism. An elevated AIP may arise from the excessive production of triglyceride-rich lipoproteins by the liver, the impaired clearance of these lipoproteins from the blood or even defects in the transport of triglycerides from the intestine by chylomicrons, indirectly influencing systemic triglyceride levels [30]. In addition, evidence suggests that high AIP values can predict coronary artery disease, heart failure and long-term myocardial infarctions [31]. These findings support previous evidence that the relationship between periodontitis, rheumatoid arthritis and cardiovascular risk may be influenced by family history and genetic predisposition, consistent with long-term clinical observations [32,33].

Undoubtedly, a major limitation of this study is the small sample size, which limited the statistical power to detect subtle but clinically relevant associations and reduces the generalizability of the findings when no statistically significant association is found. Although a power calculation was performed, it was only sufficient to detect large standardized differences, and therefore the results should be interpreted with caution and considered as preliminary or hypothesis-generating. In addition, the lack of assessment of pro-inflammatory cytokines or acute-phase reactants, apart from CRP, may have restricted our ability to further strengthen the observed associations. Future longitudinal studies are needed to evaluate the impact of periodontal interventions on lipid profiles and cardiovascular risk in vulnerable populations, such as RA patients.

In conclusion, our findings support the hypothesis that RA and periodontitis interact synergistically and are associated with a more atherogenic systemic profile. Periodontal inflammation and cumulative tissue destruction were associated with elevated LDLC, reduced HDLC, and increased atherogenic indices (CRI-1, CRI-2), suggesting that both conditions could be associated with additive effects on cardiovascular risk. This additive model highlights the importance of periodontal health as a potential modifiable factor in the cardiometabolic management of RA patients.

## Figures and Tables

**Table 1:** Patient characteristics and comparison: control vs. case (*n* = 46).

	Control (*n* = 14)	Case (*n* = 32)	
Variable	Mean ± SD	Mean ± SD	*p*-Value ^a^
Age in years (range)	37–84	15–73	0.599
Age in years	58 ± 11	54 ± 14	
Sex, n (%)			≈1
Male	3 (21.4)	8 (25.0)	
Female	11 (78.6)	24 (75.0)	
Tobacco (cig/days)			0.171 ^b^
0	14 (100.0)	28 (87.5)	
7	-	1 (3.1)	
10	-	1 (3.1)	
20	-	2 (6.3)	
BOP (%)	3.9 ± 4.0	17.0 ± 18.1	<0.001
PIRIM	0.37 ± 0.50	1.74 ± 5.27	0.281

*BOP*, Bleeding on Probing. a: Mann–Whitney’s test. b: Bilateral Fisher’s exact test for sex and tobacco.

**Table 2:** Biochemical variables: control vs. case (*n* = 46).

	Control (*n* = 14)	Case (*n* = 32)	
Variable	Mean ± SD	Mean ± SD	*p*-Value ^a^
ACPA (U/mL)	1.2 ± 0.5	112.4 ± 130.4	0.024
CRP (mg/L)	6.1 ± 6.4	8.1 ± 16.0	0.796
RF (IU/mL)	18 ± 1	115 ± 173	<0.001
Glycemia (mg/dL)	92 ± 33	88 ± 17	0.628
Cholesterol (mg/dL)	205 ± 19	207 ± 34	0.888
LDLC (mg/dL)	123 ± 19	130 ± 31	0.596
HDLC (mg/dL)	60 ± 12	61 ± 15	0.797
TG (mg/dL)	106 ± 60	86 ± 38	0.417
AIP	0.20 ± 0.23	0.12 ± 0.26	0.343
CRI-1	3.50 ± 0.68	3.59 ± 1.23	0.652
CRI-2	2.14 ± 0.62	2.27 ± 1.06	0.974
TYG	8.33 ± 0.59	8.14 ± 0.50	0.365

*ACPA*, Anti-citrullinated peptide antibody; *CRP*, C-reactive protein; *RF*, Rheumatoid factor; *TG*, Triglyceride, *AIP*, Atherogenic Index of Plasma; *CRI*, Castelli Risk Index; *TYG*, Triglyceride–Glucose Index. a: Mann–Whitney’s test.

**Table 3:** Effect of case and periodontitis ^a^ on dependent variables ^b^ (*n* = 46).

	Models with CASE and BOP (log_10_ + 1)			Models with CASE and PIRIM (log_10_ + 1)		
	Model Type I ^c^	Model Type II ^d^		Model Type I ^c^	Model Type II ^d^	
Dependent Variable	Interaction Case × BOP *p*-Value	BOP β ± se	*p*-Value	Interaction Case × PIRIM *p*-Value	PIRIM β ± se	*p*-Value
Glycemia (log_10_)	0.738	−0.02 ± 0.04	0.598	0.124	−0.01 ± 0.06	0.881
Cholesterol (log_10_)	0.053	0.05 ± 0.03	0.068	0.400	0.05 ± 0.04	0.181
LDLC (log_10_)	0.031	0.12 ± 0.03	0.001 ^e^	0.547	0.14 ± 0.05	0.018
HDLC (log_10_)	0.709	−0.07 ± 0.04	0.084	0.910	−0.18 ± 0.06	0.003
TG (log_10_)	0.418	0.04 ± 0.08	0.638	0.272	0.14 ± 0.11	0.200
AIP (log_10_ + 1)	0.425	0.03 ± 0.04	0.406	0.479	0.11 ± 0.06	0.057
CRI-1 (log_10_)	0.393	0.13 ± 0.04	0.002	0.575	0.24 ± 0.05	<0.001
CRI-2 (log_10_)	0.365	0.19 ± 0.05	0.001	0.685	0.32 ± 0.07	<0.001
TYG (log_10_)	0.748	0.00 ± 0.01	0.840	0.093	0.02 ± 0.02	0.319

*TG*, Triglyceride; *AIP*, Atherogenic Index of Plasma; *CRI*, Castelli Risk Index; *TYG*, Triglyceride–Glucose Index. a: Note that we replicate the analysis for two proxy periodontitis variables: BOP and PIRIM. b: All variables after logarithmic transformation due to asymmetry. c: Models focused on analyzing the interaction between case and BOP/PIRIM. Those models included the effect (*p*-value) of the interaction term (case × BOP/PIRIM), case and BOP/PIRIM (those single variables are not presented in the table). d: Models with no interaction between case and BOP/PIRIM. We forced those two variables, and tested age and sex (they did not enter in any studied model). We only show the effect (β and *p*-value) of BOP/PIRIM in the table. e: Since the interaction case × BOP is significant (*p* = 0.031), the stratified analysis of the association between LDLC and BOP indicates β ± se = −0.17 ± 1.76 (not significant) in controls and 2.39 ± 0.60 (*p* < 0.01) in cases.

## Data Availability

The original contributions presented in this study are included in the article/Supplementary Material. Further inquiries can be directed to the corresponding authors.

## Supplementary Materials

The following supporting information can be downloaded at: https://www.mdpi.com/article/10.3390/jcm14196722/s1, STROBE Statement—checklist of items that should be included in reports of observational studies.
